# Neuroanatomical correlates of musicianship in left-handers

**DOI:** 10.1186/s12993-024-00243-0

**Published:** 2024-06-28

**Authors:** Esteban Villar-Rodríguez, Lidón Marin-Marin, César Avila, Maria Antònia Parcet

**Affiliations:** 1 Neuropsychology and Functional Neuroimaging, Universitat Jaume I, Castelllón de la Plana, Spain; 2 Department of Psychology, University of York, York, UK; 3 York Neuroimaging Centre, Innovation Way, York, UK

**Keywords:** Musicianship, Left-handedness, Heschl’s gyrus, Arcuate fasciculus

## Abstract

**Background:**

Left-handedness is a condition that reverses the typical left cerebral dominance of motor control to an atypical right dominance. The impact of this distinct control — and its associated neuroanatomical peculiarities — on other cognitive functions such as music processing or playing a musical instrument remains unexplored. Previous studies in right-handed population have linked musicianship to a larger volume in the (right) auditory cortex and a larger volume in the (right) arcuate fasciculus.

**Results:**

In our study, we reveal that left-handed musicians (*n* = 55), in comparison to left-handed non-musicians (*n* = 75), exhibit a larger gray matter volume in both the left and right Heschl’s gyrus, critical for auditory processing. They also present a higher number of streamlines across the anterior segment of the right arcuate fasciculus. Importantly, atypical hemispheric lateralization of speech (notably prevalent among left-handers) was associated to a rightward asymmetry of the AF, in contrast to the leftward asymmetry exhibited by the typically lateralized.

**Conclusions:**

These findings suggest that left-handed musicians share similar neuroanatomical characteristics with their right-handed counterparts. However, atypical lateralization of speech might potentiate the right audiomotor pathway, which has been associated with musicianship and better musical skills. This may help explain why musicians are more prevalent among left-handers and shed light on their cognitive advantages.

**Supplementary Information:**

The online version contains supplementary material available at 10.1186/s12993-024-00243-0.

## Background

Approximately 10.6% of the human population is estimated to be left-handed [1]. Popular belief associates left-handedness with a unique talent for composing and interpreting music. This notion arises from the fact that some of the world’s most renowned musicians have exhibited left-handed playing [2], along with the popularity of scientific accounts postulating the idea of ‘gifted’ left-handers [3, 4]. Contrary to this, an initial observation by Oldfield [5] suggested that left-handedness might hinder the ability to play a musical instrument, given that most instruments are designed for right-handed individuals. However, subsequent reports on student samples have failed to establish a negative association between left-handedness and musical aptitude [5, 6]. In line with common belief, most studies have revealed a higher prevalence of left-handedness in musicians compared to non-musicians among both professionals [7, 8] and students [9, 10]. Additionally, an increase in the proportion of mixed-handers among musicians was also noted [11]. Furthermore, some publications have highlighted that left-handed musicians excel in certain musical skills in comparison to their right-handed counterparts [12, 13].

The explanation for this phenomenon may reside in the unique characteristics of the musicians’ and left-handers’ brains. Musicianship has been related to structural changes in the auditory cortex, particularly in Heschl’s gyrus (HG) [14, 15]. Various studies have reported that musicians present increased gray matter volume in the bilateral HG [16] and the left HG [17]. But the great majority have observed these differences in the right HG, both cross-sectionally [18–20] and longitudinally in relation to musical training [21–23]. Moreover, gray matter volume in HG has been shown to predict better performance on pitch discrimination tasks [24, 25].

However, it is important to note that all the aforementioned results originate from right-handed musicians. When considering the left-handed brain, it is important to note that cortical asymmetries across all lobules have been reported to be altered [26, 27]. Therefore, the association between musicianship and its related structural changes — seemingly favoring the right hemisphere — might differ among left-handers. Right-handed individuals typically exhibit a significant leftward asymmetry in the auditory cortex [28–32], but data on the left-handed population is inconsistent. Some studies focusing on the auditory cortex have described a reduction in this leftward asymmetry among left-handed participants [33–35]. This reduction, however, has not been replicated in multicentric studies comparing large groups of left-handed and right-handed individuals [27, 36, 37]. In this context, atypical dominance for language should also be considered [38], because of: (1) its proposed relation with altered auditory asymmetries in both healthy [28, 39–41] and clinical populations [42–46]; and (2) its higher incidence among the left-handed population [38, 47].

A second brain structure that has been significantly implicated in musicianship is the arcuate fasciculus (AF). This white matter tract connects the posterior part of the superior temporal gyrus with the parietal cortex and the inferior frontal gyrus, supporting functions like language, music processing and motor planning [48]. Previous studies have revealed that musicians, when compared to non-musicians, exhibit a larger volume and greater white matter integrity in the right AF [49, 50]. Moreover, early musical training has been shown to increase the volume of the right AF, reducing its typically leftward asymmetry [51]. Different research also demonstrates the relationship between this white matter fascicle and music processing capabilities. First, congenital and acquired amusia — the inability to recognize and reproduce tones and rhythms — has been associated with alterations in the right AF [52–54]. Also, the structural characteristics of the right AF seem to predict melody and rhythm learning [55] and pitch-related grammar learning [56].

The AF, like the HG, presents a significant structural asymmetry in the healthy population. Its direct segment — connecting the temporal and frontal cortices — is larger in the left hemisphere [57], whereas its anterior segment — bridging the parietal and frontal cortices — is larger in the right hemisphere [58]. Unfortunately, there are few reports on the possible differences in the AF between right-handed and left-handed individuals. Tractography studies have mostly failed to find any neuroanatomical signature of handedness [59], suggesting that differences may be attributed to hemispheric lateralization of language [60, 61] — although subsequent studies have also failed to find this association [62–64] — or to the degree of handedness rather than its direction [65]. However, a large study on the UK Biobank identified a common genetic basis for handedness and structural development of the AF, hinting at a potential correlation [66]. Therefore, it is unclear that AF presents differences according to handedness.

Taken altogether, these findings provide valuable insights into the neuroanatomical aspects of musicianship, its overlapping with left-handedness, and the possible underlying role of hemispheric language dominance. A greater development of certain components of the right audiomotor network — HG and AF — seems to facilitate music processing capabilities. However, left-handedness — and its associated atypical language lateralization — have also been partially related with neuroanatomic differences in these structures. This begs the question of how these musicianship correlates operate on the left-handed brain, as both its structural peculiarities and the higher incidence of left-handedness among professional musicians may suggest a change in the direction of the effects. Unfortunately, brain research in left-handed musicians is almost nonexistent [41]. Hence, further investigation is needed to better comprehend the potentially complex relationship between handedness, musicianship, and cognitive functions. The present study was designed with this objective in mind. The volume of the HG and the architecture of the AF were explored using MRI in left-handers as a function of both musicianship (musicians vs. non-musicians) and hemispheric lateralization of speech (typically vs. atypically lateralized during a verb generation task). Given the previously presented evidence, we predict that handedness should not impact musicianship-related changes to cerebral structure [18–20, 49–51, 55, 56]. But atypical language lateralization, which underlies 22–27% of left-handers [38, 47], might be a relevant factor in this music-structure interplay [28, 39–41, 63]. Therefore, we hypothesize that: (1) musicianship among left-handers would be related to structural differences in the HG and the right AF, similar to previous studies among right-handers; and (2) that the hemispheric direction or the magnitude of these differences would be modulated by the underlying hemispheric lateralization of speech.

## Methods

### Participants

A total of 130 healthy left-handed (*n* = 109) and mixed-handed (*n* = 21) individuals according to the Edinburgh Handedness Inventory (EHI) [67] participated in the study. EHI was scored according to the recommendations by Bryden [68], ranging from 24 to 37 (mixed-handed) to 38–50 (left-handed). All participants were capable of writing exclusively with their left hand. 55 were musicians [27 women; mean ± SD age = 23.5 ± 5.7 years; mean EHI ± SD = 43.3 ± 5.5] and 75 were non-musicians (40 women; mean ± SD age = 22.9 ± 5.6 years; mean EHI ± SD = 42.9 ± 4.6). Musicians had completed musical training at a music school, and were currently playing a musical instrument (mean ± SD age of onset of training = 7.8 ± 2.4 years, range = 3–15; minimum of 6 years of formal training). Non-musicians had never played a musical instrument nor received musical training beyond obligatory musical instruction at school. The two groups did not significantly differ in age (*t*_*128*_ = 0.596; *P* = .552), gender distribution (Pearson’s *χ*^*2*^ = 0.229; *P* = .633) or EHI score (*t*_*128*_ = 0.47; *P* = .639). None of the participants had suffered from any neurological or psychiatric disorders, and they had no history of head injury with loss of consciousness. Written informed consent was obtained from all participants, following a protocol approved by the Universitat Jaume I, and they received monetary compensation for their participation.

### Image acquisition

Magnetic resonance images (MRI) were acquired on a 3T GE Signa-Architect scanner using a 32-channel head coil. All slices were acquired in the sagittal plane. A 3D structural MRI was acquired for each subject using a T1-weighted magnetization-prepared rapid gradient-echo sequence (TR/TE = 8.5/3.3 ms; flip angle = 12; matrix = 512 × 512 × 384; voxel size = 0.47 × 0.47 × 0.5 mm). Gradient-echo T2*-weighted echo-planar imaging sequences were recorded during a verb generation task (functional volumes = 144; TR/TE = 2500/30 ms; flip angle = 70; matrix = 64 × 64 × 30; voxel size = 3.75 × 3.75 × 4 mm). A diffusion-tensor-imaging (DTI) sequence was also acquired (diffusion gradient directions = 25, b = 0 + 1000 mm^2^/s, TR/TE = 13,000/80; flip angle = 90; matrix = 256 × 256 × 60; voxel size = 1.01 × 1.01 × 2 mm).

### Verb generation task

#### Task design

We used a computerized Spanish verb generation task suited for MRI scanners, that is described elsewhere [41, 69]. In summary, it consists of an activation condition (6 blocks, 30 s each) in which the participant generates verbs from visually presented concrete nouns, and a control condition (6 blocks, 30 s each) in which the participant reads aloud visually presented pairs of letters. Stimuli were presented using MRI-compatible goggles (VisuaStimDigital, Resonance Technology Inc.), and responses were recorded via a noise-cancelling microphone to ensure that each participant was actively and accurately engaged in the task (FOMRI III+, Optoacoustics). Before entering the scanner, participants practiced with a different version of the task for one minute.

#### fMRI preprocessing

Functional images acquired during the verb generation task were processed using the Statistical Parametric Mapping software package (SPM12; Wellcome Trust Centre for Neuroimaging, London, UK). Preprocessing followed the default pipeline and included: (1) alignment of each participant’s fMRI data to the AC-PC plane by using the anatomical image; (2) head motion correction, where the functional images were realigned and resliced to fit the mean functional image; (3) co-registration of the anatomical image to the mean functional image; (4) re‐segmentation of the transformed anatomical image using a tissue probability map; (5) spatial normalization of the functional images to the MNI (Montreal Neurological Institute, Montreal, Canada) space with 3 mm3 resolution; and (6) spatial smoothing (FWHM = 4 mm). A general linear model was voxel-wise defined for each participant by contrasting ‘activation > control’ volumes. The BOLD (Blood‐Oxygen‐Level‐Dependent) signal was estimated by convolving the block’s onsets with the canonical hemodynamic response function. Six motion realignment parameters extracted from head motion preprocessing were included as covariates of no interest, and a high‐pass filter (128 s) was applied to the contrast images to eliminate low‐frequency components.

#### Assessment of hemispheric lateralization

Individual functional lateralization of speech was assessed by calculating the Laterality Index (LI) for the verb generation contrast images resulting from the prior preprocessing. LI is a proportion of the brain activation between the two hemispheres, thus giving us information about the direction and degree of hemispheric specialization during a particular function in a single individual. LI was calculated according to the formula (L − R/R + L) × 100, where L corresponds to the number of significantly active voxels in the left hemisphere mask, and R corresponds to the number of significantly active voxels in the right hemisphere mask. Using this formula, LI ranges from + 100 (totally leftward function) to − 100 (totally rightward function). We computed a weighted mean LI for every participant using the bootstrap method implemented in the LI-toolbox [70], based on SPM. We computed the LI encompassing the frontal voxels most relevant in the expressive language function evaluated by this task. We defined this using standard anatomical criteria [71], thus including Brodmann areas 9, 44, 45 and 46. We created this mask via the Tailarach Daemon atlas [72] included in the WFU PickAtlas toolbox [73], inserting the Brodmann areas with a 3D dilatation value of 2. Additionally, medial areas were subtracted by a boxcar with dimensions of 20, 100, 100, and an epicenter at 0, 0, 0. Speech production was subject-wise classified as typically lateralized (LI higher than + 40) or atypically lateralized (LI lower than + 40). We used + 40 as a cut-off point (contrary to the more traditional + 20), based on previous findings that emphasized the importance of lateralization strength when grouping individuals [38, 74]. Averaged activation maps for the typical and atypical groups can be found in the Supplementary Material.

### Voxel-based morphometry

3D structural MRI images were preprocessed using a voxel-based morphometry (VBM) approach, via the CAT12 toolbox (http://www.neuro-jena.github.io/cat/) for SPM12. Preprocessing followed the default pipeline and included: (1) segmentation into gray matter, white matter, and cerebrospinal fluid; (2) registration to the ICBM standard template; and (3) DARTEL normalization of gray matter and white matter segments to the MNI template. After that, we extracted region of interest (ROI) native gray matter volume values corresponding to the primary auditory cortex or HG (Brodmann areas 41 and 42). Additionally, structural LIs were computed for these gray matter volumes in each participant, using the same formula described for the verb generation task.

### Diffusion-based tractography

DTI images were corrected for eddy current distortions using FSL [75]. After that, we followed the default pre-processing pipeline of DiffusionToolkit [76] to reconstruct the images, track the fibers and smooth the tracks. Fractional anisotropy was used for the masking procedure during fiber tracking (threshold = 0.2), setting the angle threshold at 35º. Virtual dissection of the arcuate fasciculus (AF) followed the guidelines provided by previous tractography studies [77, 78], using the TrackVis software (trackvis.org). In summary, dissection was carried out for all participants in a single-subject basis by displaying their track reconstruction in native space and performing manual region of interest (ROI) delineations to encompass the AF segments in both hemispheres. The anterior AF segment was delineated by drawing a coronal prefrontal ROI and a sagittal inferior parietal ROI. The posterior AF segment was delineated by drawing a sagittal inferior parietal ROI and an axial superior temporal ROI. The direct AF segment was delineated by drawing a coronal prefrontal ROI and an axial superior temporal ROI. Number of streamlines was extracted for every AF segment from each participant. Additionally, structural LIs were computed for these number of streamlines in each participant, using the same formula described for the verb generation task.

### Statistical analyses

We conducted analyses of structural measures using various repeated-measures MANOVA designs. Note that all designs included total intracranial volume and age as covariates of no interest, except for designs involving structural LIs. We examined the potential impact of musicianship (musician/non-musician) on gray matter volume in HG, with hemisphere (left/right) considered as a within-subject variable. We studied HG structural LI when comparing musicians and non-musicians via an ANOVA. For the diffusion data, we also explored the potential effect of musicianship (musician/non-musician) on AF number of streamlines, with hemisphere (left/right) and segment (anterior/posterior/direct) considered as within-subject variables. AF structural LIs were included as dependent variables in a repeated-measures ANOVA contrasting musicians and non-musicians. In all designs, we further investigated whether the introduction of speech lateralization as a factor (typical/atypical) altered the potential effects of musicianship. Significant interactions were interpreted via post-hoc pair-wise Bonferroni-corrected *t* tests. Post-hoc, we also conducted Spearman’s correlations to determine whether several covariates of musical training (age of training onset, years of training, and estimated lifetime training hours) had a significant impact on both the bilateral HG volume and the number of streamlines in the right anterior AF segment among musicians. All statistical analyses were performed using IBM SPSS Statistics version 26, with no custom scripts involved.

## Results

**Fig. 1:**
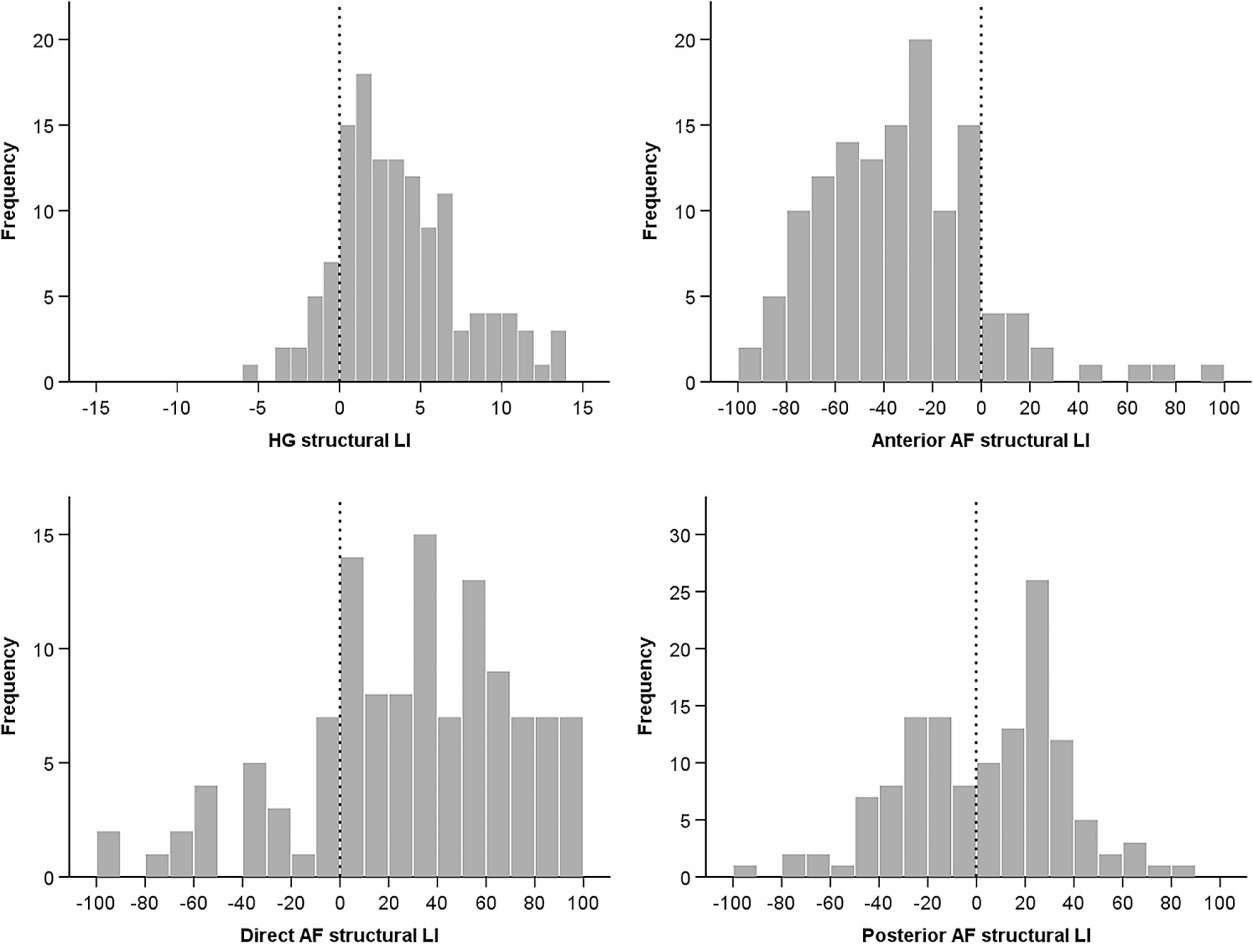
Histograms displaying the structural LI (Laterality Index) for the HG (Heschl’s gyrus), and the different segments of the AF (arcuate fasciculus). Positive values correspond to leftward asymmetry, whereas negative values indicate rightward asymmetry

Table 1 displays estimated means and standard errors of gray matter volume in Heschl’s gyri (HG) based on musicianship and speech lateralization groups, as well as averaged structural LIs. Figure 1 depicts the distribution of structural LIs for the sample. Note the slightly leftward structural asymmetry present in most participants.

**Table 1:** Estimated mean (standard error) values for the gray matter volume of the left and right Heschl’s gyri (HG), grouped by musicianship and speech lateralization

**Volume (ml)** ^**a**^	**Non-musicians (** ***n*** ** = 75)**		**Musicians (** ***n*** ** = 55)**	
Left HG	5.01 (0.06)		5.21 (0.06)	
Right HG	4.60 (0.06)		4.87 (0.06)	
Structural LI^**b**^	4.1 (3.8)		3.3 (3.7)	
**Volume (ml)** ^**a**^	**Typical (** ***n*** ** = 56)**	**Atypical (** ***n*** ** = 19)**	**Typical (** ***n*** ** = 34)**	**Atypical (** ***n*** ** = 21)**
Left HG	5.01 (0.06)	4.99 (0.1)	5.19 (0.07)	5.22 (0.09)
Right HG	4.62 (0.06)	4.57 (0.1)	4.89 (0.07)	4.86 (0.09)
Structural LI^**b**^	4 (4.1)	4.4 (3.1)	3.1 (3.2)	3.6 (4.5)

^a^Values are estimated for a mean age of 23.2 years and a mean total intracranial volume of 1507.77 ml

^b^Averaged structural LI (standard deviation). Positive values indicate leftward asymmetry, whereas negative values indicate rightward asymmetry

**Fig. 2:**
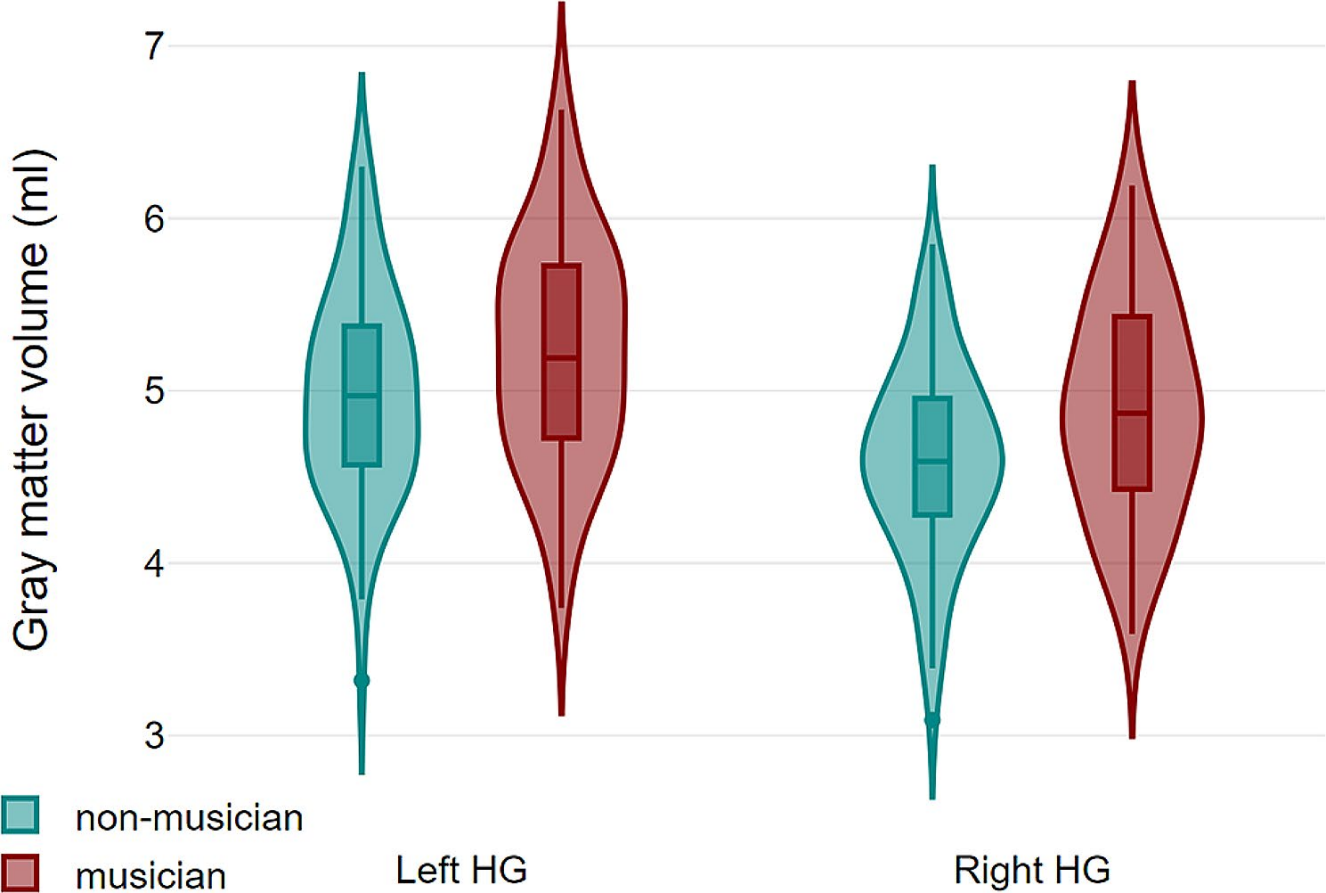
Violin plots displaying the gray matter volume of left HG and right HG in non-musicians (green) and musicians (red). Presented values are unadjusted for age and total intracranial volume

We conducted a repeated-measures multivariate analysis of variance, using HG gray matter volumes as dependent variables, hemisphere as a within-subjects factor, and musicianship as between-subjects factor. Total intracranial volume and age were included as covariates of no interest. Results showed a significant main effect for musicianship (*F*_*126*_ = 11.7; *P* < .001; *η*^2^ = 0.085), indicating larger HG volume in musicians compared to non-musicians across both hemispheres (Fig. 2). No significant effect (*F*_*126*_ = 0.38; *P* = .54) nor interaction with musicianship (*F*_*126*_ = 1.11; *P* = .293) was found involving hemisphere. Notably, HG volume did not significantly vary across musicians in relation to their age of training onset (⍴ = 0.021; *P* = .884), years of training (⍴ = 0.037; *P* = .793), or estimated lifetime training hours (⍴ = 0.062; *P* = .658). The addition of speech lateralization as between-subject factor did not alter these results. Therefore, left-handed musicians exhibit increased bilateral HG gray matter volume regardless of hemispheric language dominance.

We also explored the asymmetry of HG using a similar analysis of variance. We included HG structural LI as dependent variable, and musicianship as between-subjects factor. However, no significant effect was found for musicianship (*F*_*129*_ = 1.5; *P* = .223). The addition of speech lateralization as between-subject factor did not alter this result. Thus, structural asymmetry of the HG does not seem to be influenced by either musicianship or hemispheric language dominance.

Table 2 presents estimated means and standard errors of white matter streamlines in the three segments of the arcuate fasciculi (AF), categorized by musicianship and speech lateralization groups, as well as averaged structural LIs. Figure 1 depicts the distribution of structural LIs for the sample. Note the important leftward structural asymmetry of the direct AF, in contrast to the rightward asymmetry of the anterior AF.

**Table 2:** Estimated mean (standard error) values for the number of white matter streamlines in the left and right segments of the arcuate fasciculi (AF), grouped by musicianship and speech lateralization

**Number of streamlines** ^**a**^	**Non-musicians (** ***n*** ** = 75)**		**Musicians (** ***n*** ** = 55)**	
Left Direct AF	499 (39)		511 (39)	
Left Anterior AF	464 (46)		431 (48)	
Left Posterior AF	890 (57)		921 (60)	
Right Direct AF	359 (41)		277 (42)	
Right Anterior AF	843 (64)		1076 (66)	
Right Posterior AF	850 (63)		909 (65)	
Structural LI Direct AF^**b**^	29.4 (47.8)		40.8 (43.6)	
Structural LI Anterior AF^**b**^	—26 (34.9)		—43.8 (27.3)	
Structural LI Posterior AF^**b**^	4.6 (32.1)		0.3 (34.4)	
Structural LI AF (averaged)^**b**^	2.7 (23.2)		—0.9 (18.7)	
**Number of streamlines** ^**a**^	**Typical (** ***n*** ** = 56)**	**Atypical (** ***n*** ** = 19)**	**Typical (** ***n*** ** = 34)**	**Atypical (** ***n*** ** = 21)**
Left Direct AF	479 (38)	518 (65)	535 (49)	487 (62)
Left Anterior AF	599 (46)	330 (80)	479 (60)	383 (76)
Left Posterior AF	1001 (57)	779 (99)	902 (75)	941 (95)
Right Direct AF	275 (41)	443 (70)	249 (53)	306 (67)
Right Anterior AF	966 (64)	721 (110)	1077 (83)	1076 (106)
Right Posterior AF	876 (63)	823 (109)	910 (82)	907 (104)
Structural LI Direct AF^**b**^	35.9 (47.2)	10.2 (45.7)	47.3 (38.3)	30.2 (50.2)
Structural LI Anterior AF^**b**^	—22.6 (33.2)	—36.1 (38.8)	—39.9 (26.7)	—50 (27.9)
Structural LI Posterior AF^**b**^	6.7 (32.2)	—1.6 (31.6)	—1.8 (29.9)	3.6 (41.2)
Structural LI AF (averaged)^**b**^	6.7 (21.6)	—9.1 (24.3)	1.8 (16.2)	—5.4 (21.8)

^a^Values are estimated for a mean age of 23.2 years and a mean total intracranial volume of 1507.77 ml

^b^Averaged structural LI (standard deviation). Positive values indicate leftward asymmetry, whereas negative values indicate rightward asymmetry

**Fig. 3:**
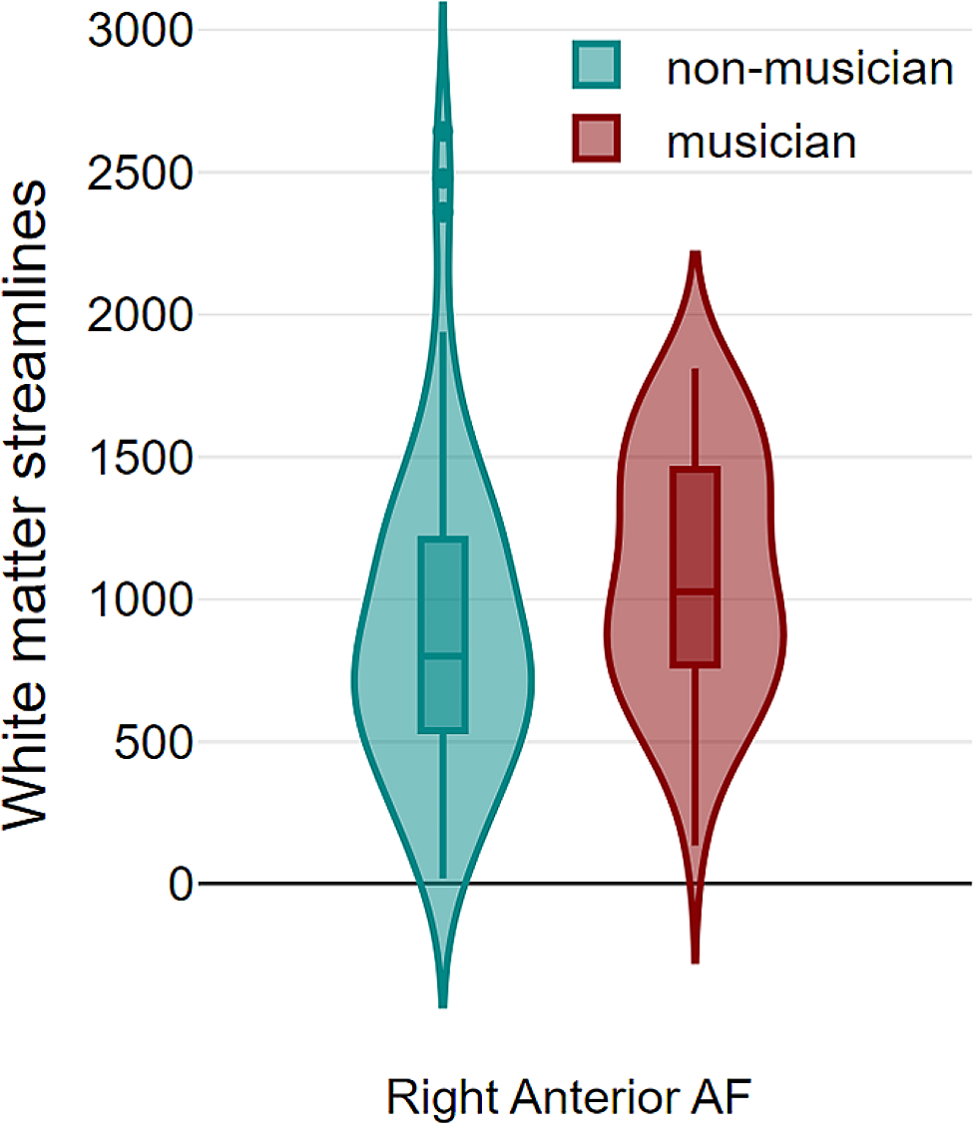
Violin plots displaying the number of white matter streamlines across the right anterior AF segment in non-musicians (green) and musicians (red). Presented values are unadjusted for age and total intracranial volume

We conducted a repeated-measures multivariate analysis of variance, utilizing AF white matter streamlines as dependent variables, with hemisphere plus segments as within-subject factors, and musicianship as between-subjects factor. Total intracranial volume and age were included as covariates of no interest. A significant hemisphere × segment × musicianship interaction revealed a higher number of streamlines in the right anterior segment of the AF among musicians (*F*_*125*_ = 4.26; *P* = .016; *η*^2^ = 0.064; pair-wise Bonferroni-corrected *P* = .048) (Fig. 3). Additionally, it should be noted that the hemisphere × musicianship interaction was close to significance (*F*_*126*_ = 3.31; *P* = .071; *η*^2^ = 0.026), suggesting a tendence towards a rightward AF in musicians, independently of segments. The main effect of hemisphere was not significant (*F*_*126*_ = 0.57; *P* = .452). Notably, the number of streamlines in the right anterior segment did not significantly vary across musicians in relation to their age of training onset (⍴ = −0.01; *P* = .951), years of training (⍴ = −0.225; *P* = .105), or estimated lifetime training hours (⍴ = −0.142; *P* = .311). Introduction of speech lateralization as a between-subject factor did not alter any effect. Consequently, left-handed musicians present a larger right anterior AF, irrespective of hemispheric language dominance.

Finally, we explored the asymmetry of the different segments of AF using a similar analysis of variance. We included AF structural LIs as dependent variables, defining segment as within-subject factor, and musicianship as between-subjects factor. A significant segment × musicianship interaction was found (*F*_*127*_ = 4.45; *P* = .014; *η*^2^ = 0.065), indicating that musicians presented a more marked rightward asymmetry of the anterior AF than non-musicians (pairwise Bonferroni-corrected *P* = .002). This aligns with the increased number of streamlines in the right segment among musicians. Interestingly, the inclusion of hemispheric language dominance resulted in a main effect (*F*_*126*_ = 8.36; *P* = .005; *η*^2^ = 0.062), revealing that typicals averaged a leftward asymmetry of the full AF (mean + SD = 4.6 + 19.7), whereas atypicals showed an opposite rightward pattern (− 7.2 + 22.8).

## Discussion

The current study investigates whether left-handedness — and its corresponding variability in hemispheric language lateralization — might impact the well-established association between musicianship and the neuroanatomic characteristics of Heschl’s gyrus (HG) and the arcuate fasciculus (AF). Mirroring the observations from studies involving right-handed individuals, our findings reveal that left-handed musicians, when compared to non-musicians, display increased gray matter volume in both left and right HG, alongside a greater number of streamlines in the anterior segment of the right AF. These differences remained uninfluenced by the hemispheric lateralization of speech. But notably, atypically lateralized individuals (both musicians and non-musicians) presented a rightward structural asymmetry of their full AF, in contrast to the leftward asymmetry exhibited by typicals. Consequently, left-handed musicians demonstrate a similar pattern of cerebral differences as previously noted in right-handed musicians, but their greater development of the right AF could be potentiated by an atypical language dominance.

Despite the historical notion of left-handedness posing a challenge in playing bimanual instruments [5], evidence indicates that the prevalence of left-handed musicians is normal or even higher compared to right-handed musicians [6–11]. Thus, specific factors might be facilitating the engagement of left-handed individual in musical pursuits. One possibility is that the cerebral organization of the left-handed brain confer advantages for musical processing. In this sense, some studies have reported that left-handedness is associated with better performance in musical-related skills such as pitch perception [79], pitch memory [80], sight reading [13], or even a higher aptitude for music [12]. In the same vein, left-handers also perform better in motor tasks requiring intermanual coordination [81] and the use of their non-dominant hand [82, 83]. Relatedly, a single-case report suggested that left-handed musicians exhibit greater adaptability when adjusting their performance to changes in internal motor representations [84].

Our results reveal that left-handed musicians show the same neuroanatomical characteristics as their right-handed counterparts, at least in the auditory cortices and audiomotor pathways. The whole left-handed sample, irrespective of musicianship, replicated the leftward asymmetry of the HG [28–32], the leftward asymmetry of the direct AF [61], and the rightward asymmetry of the anterior AF [58]. Regarding musicianship, it has been linked with an increased gray matter volume in the right auditory cortex [18–23], left auditory cortex [17], or both auditory cortices [16]. Crucially, we observed this effect on both HG when comparing left-handed musicians and non-musicians. The size and integrity of the anterior AF — particularly on the right hemisphere — has been associated with instrumentalist training [49, 51, 85], musical learning [55], and amusia [52–54]. Accordingly, we found a higher number of streamlines — a measure closely related to tract volume — in the right anterior AF of left-handed musicians, but no other diffusion-based measure demonstrated significant differences in these fasciculi. Although atypical hemispheric lateralization of speech — established using fMRI [38, 41, 47] — was present in 38% of musicians and 25% of non-musicians, it did not modify the relationship between musicianship and HG volume or AF streamlines in left-handers. In other words, musicians with atypical language dominance also presented larger bilateral HG and right anterior AF. However, we revealed that individuals with atypical lateralization exhibited a rightward asymmetry in their AF (all segments considered), which significantly differed from the leftward asymmetry shown by typically lateralized (irrespective of musicianship). This result aligns with a previous report on an atypically lateralized child [60] and is, to our knowledge, the first supporting evidence in an adult sample for the proposal that AF asymmetry might underlie language lateralization [61]. It should be noted that previous studies have failed to find this association [62–64]. Thus, we cannot discard the possibility that this effect was detected due to the high proportion of musicians in our sample. Nevertheless, according to our data, atypical lateralization of language could have an influence on the audiomotor pathway, potentially summing to the structural differences favoring the right AF that have been associated with improved musical processing [55, 56] and musicianship [49, 50]. Crucially, this effect of atypical language dominance in AF asymmetry resembles the previously reported impact of early musical training [51].

Apart from this, we should consider that the potential advantageous characteristics of left-handedness might also reside in other brain regions. A recent large-scale study confirmed that left-handedness is associated with increased rightward asymmetries and decreased leftward asymmetries in the motor and premotor cortices involved in hand control [27]. Moreover, research indicates that bimanual motor coordination is mainly controlled by the (hand) dominant hemisphere [86], and that left-handers recruit a more bilateral functional network when performing or imagining a hand motor task [87]. Collectively, these findings suggest that differences related to musicianship might be potentiated by atypical language dominance, which combined with a reversed or altered organization of motor areas (due to left-handedness), might underlie cognitive differences facilitating left-handers into musicianship. Future studies should explore these possibilities.

## Conclusions

This is the first time that the neuroanatomical correlates of musicianship have been explored in left-handed population. In summary, left-handed musicians exhibited increased volumes in the bilateral auditory cortex and a greater number of streamlines traversing the right arcuate fasciculus. These traits remain unaffected by the presence of atypical language dominance, but this rare phenomenon was associated to a rightward shift in the structural asymmetry of the arcuate fasciculus. Consequently, the observed pattern in left-handed musicians was not different from that previously identified in right-handed musicians, but it may be potentiated by the presence of atypical lateralization of speech. Thus, the higher prevalence of left-handers among musicians may respond to the higher incidence of atypical lateralization of language among the left-handed population. Future research should explore whether the co-occurrence of manual dominance and music processing within the same hemisphere also leads to variations in instrumental performance.

### Electronic supplementary material

Below is the link to the electronic supplementary material.

Supplementary Material 1

## Data Availability

The datasets generated and analysed during the current study are available in the figshare repository, 10.6084/m9.figshare.24866709.v1.
