# Supplementary material for: Neuroanatomical correlates of musicianship in left-handers
Source: Behav Brain Funct. 2024 Jun 28;20:17. doi: 10.1186/s12993-024-00243-0 (PMC11214256; doi:10.1186/s12993-024-00243-0)
Supplement: Supplementary file 1 — Supplementary Material 1 [file 12993_2024_243_MOESM1_ESM.docx]

# SUPPLEMENTARY MATERIAL


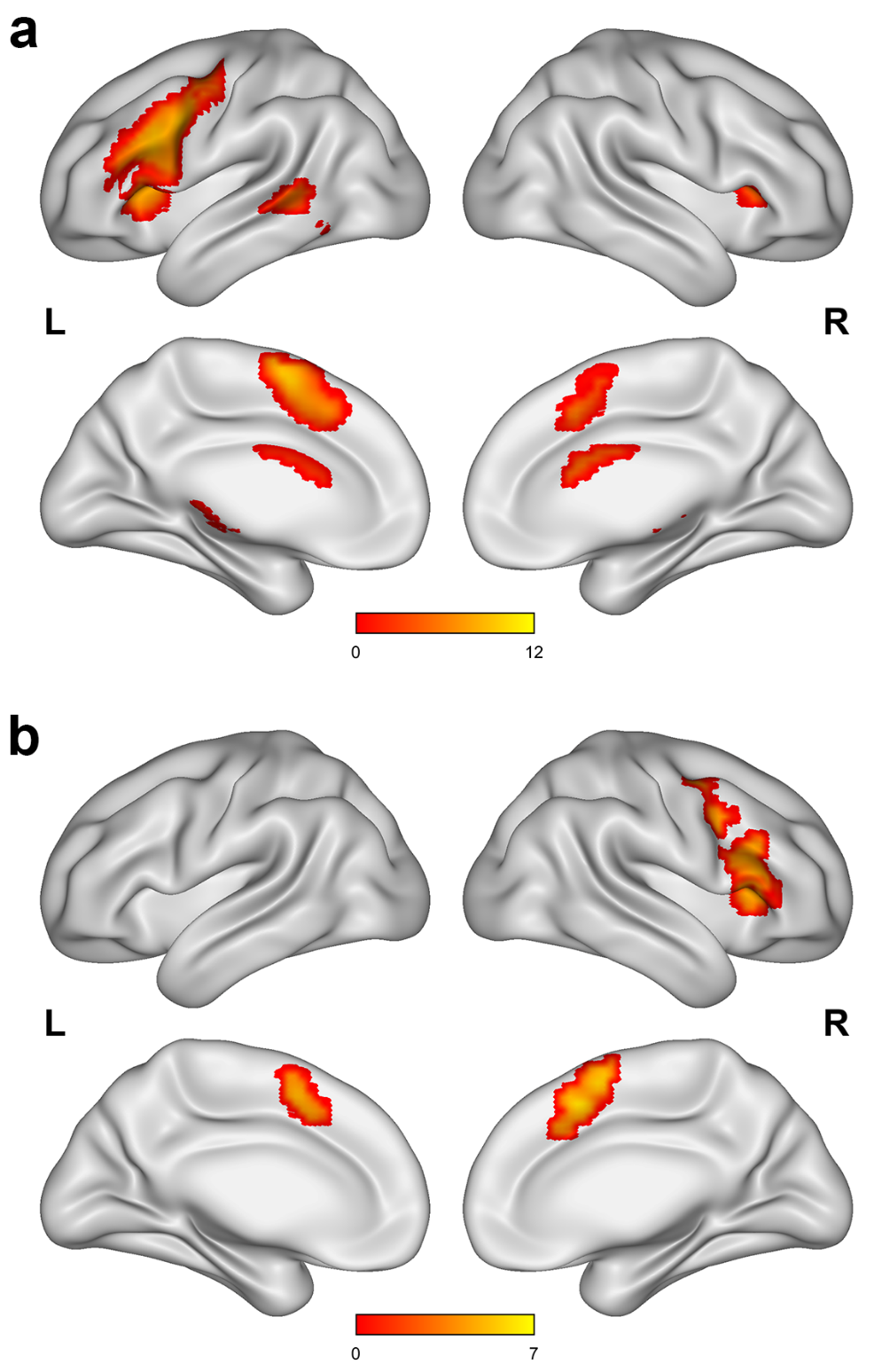


**Supplementary Figure 1.** Activation maps during the verb generation task. (**a**) typically lateralized group. (**b**) atypically lateralized group. Voxel-wise one-sample *t* tests for ‘activation > control’ condition (*P* = .001, FWE-cluster corrected at *P* < .05). The color bar represents *t* value. L = left hemisphere, R = right hemisphere.


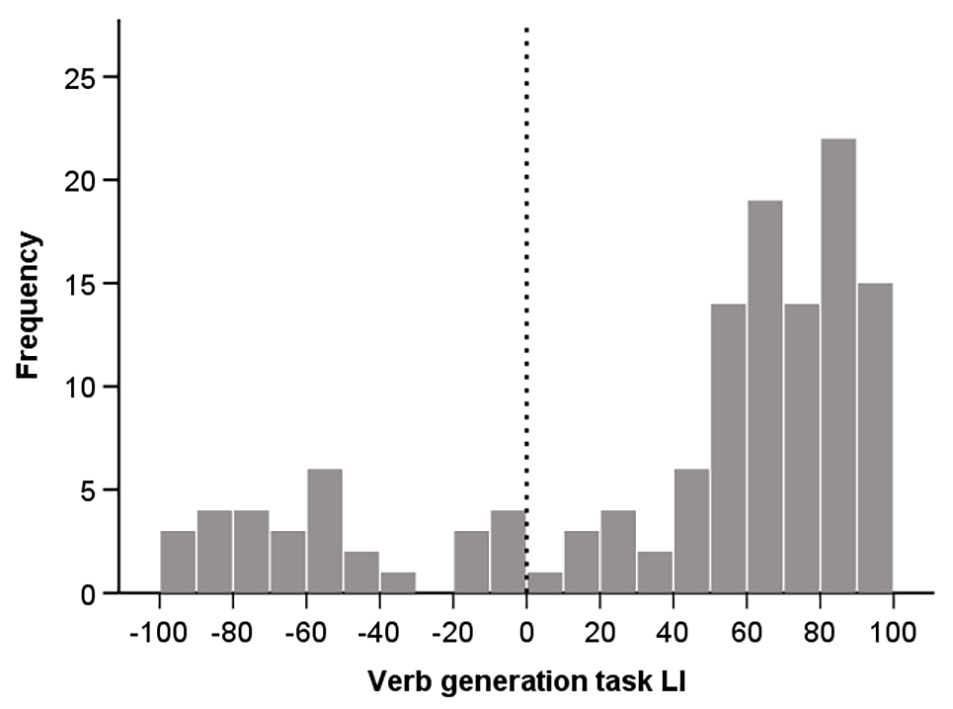


**Supplementary Figure 2.** Histogram displaying the verb generation task LI (Laterality Index). Positive values correspond to leftward functional asymmetry, whereas negative values indicate rightward functional asymmetry.
